# 

**Published:** 1920-01-17

**Authors:** 


### Purchase of an Infirmary.

THE Metropolitan Asylums Board has approved the purchase of the Hendon Infirmary from the City of Westminster Guardians, at a price of £160,000, for the accommodation of tuberculous patients.

### Gifts and Bequests.

The King has sent £10 to the Great Northern Central Hospital.

Mrs. Annie Goff, of Upper Norwood, made a large number of bequests, including £3,000 to the Hospital for Sick Children for the endowment of cots, £2,000 to the Florence Nightingale Hospital for Gentlewomen, and £1,000 to St. Dunstan's. Subject to numerous other legacies, the residue of her property is to be divided between a number of charitable institutions. Amongst them are the Mineral Water Hospital, Bath; Children's Hospital, Bath; the Foundling Hospital; the Cripples' Home at Alton; Guy's Hospital; St. Bartholomew's Hospital; Middlesex Hospital; Cancer Hospital; Soho Square Hospital for Women; Queen Charlotte's Hospital; Skin Hospital, Blackfriars Road; Paddington Green Children's Hospital; Temperance Hospital; London Fever Hospital; London Throat Hospital; St. Saviour's Hospital; and the Lock Hospital.

The Chelsea Hospital for Women has received £100 from the Alexandra Day Fund and £900 bequest from the late Mr. A. H. Lloyd.

The Royal Edinburgh Hospital for Sick Children will receive £500 and the County and City Infirmary, Perth, £200 under the will of the late Mr. John Mackay Bernard, B.Sc., F.R.S.E., of Dunsinane, Perthshire.

The Committee of Alexandra Day have sent £350 to Queen Charlotte's Hospital and £200 to the Royal Hospital and Home for Incurables, Putney.

Huddersfield Royal Infirmary has received from Messrs. John Brooke and Sons £2,000 to endow a bed in memory of the men who worked at Armitage Bridge Mills and gave their lives during the war.

Mr. Frederick Burgoyne, of Harley Street, W., left £1,000 each to the Middlesex Hospital and St. Bartholomew's Hospital. The residue of the estate will ultimately be distributed amongst charitable objects, at the discretion of the executors.

Under the will of the late Mr. Frederick Knight, of Castle Bromwich, Birmingham, hospitals will benefit as follows: General Hospital, £500; Hospital for Women, the Eye Hospital, and the Ear and Throat Hospital, £250 each. The Royal Orphanage, Wolverhampton, is also left £250.

Mr. Jesse Tildesley, of Willenhall, has left £50 to the Wolverhampton and Staffordshire Hospital and £20 to the Willenhall Nursing Association.

Grimsby Hospital has been left £100 by the late Mr. G. W. Goddard, a shipowner of Great Grimsby.

The residue of the estate of the late Mr. W. H. L. Ray, of Worthing, has been bequeathed to Earlswood Asylum for Idiots. It is expected that the sum receivable will be about £55,000.

The Royal Victoria Hospital, Belfast, has received a gift of £500 from Mr. and Mrs. R. W. McDorrell to name a bed.

### Passing Events and Topics.

### The Association of Certificated Blind Masseurs.

The Association under the above name has been formed to look after the interests of blinded soldiers who have been taught the occupation of massage. The address is 224-228 Great Portland Street.

### Brighton and Hospital Sunday.

The Brighton, Hove, and Preston Hospital Sunday Fund has raised this year the biggest total—£1,537—since 1888.

### Royal Sanitary Institute.

The Right Hon. Viscount Astor has consented to accept the office of President of the 31st Congress of the Royal Sanitary Institute, to be held at Birmingham from July 19 to 24, 1920.

### From Lips of Pain.

The Christmas appeal of the Royal Hospital and Home for Incurables, Putney, took the form of an attractive booklet, illustrated by "Chris," and written, we suspect, by the Secretary, Mr. Charles Cutting.

### Midwives' Training Regulations, 1919.

The Board of Education announces that the Draft, dated September 19, 1919, of the Regulations for the Training of Midwives has been published for the required period, and has now been confirmed by the Board without amendment. The Draft now becomes "The Board of Education (Midwives' Training) Regulations, 1919," dated November 24, 1919, and copies can be purchased through any bookseller (price 1d.) or directly from H.M. Stationery Office, Imperial House, Kingsway, London, W.C. 2 (price by post 1½d.).

### MATRONS' AND SISTERS' APPOINTMENTS.

Royal Asylum, Aberdeen.—Miss Elspeth MacRae has been appointed assistant matron. She was trained at the Royal Infirmary, Aberdeen, has been sister at the Morningfield Hospital, Aberdeen, and military nursing sister attached to the 1st Scottish General Hospital, Aberdeen.

Worcester General Infirmary.—Miss M. F. Watson has been appointed matron. She was trained at Bolton General Infirmary, has been sister-in-charge at the Monsall Fever Hospital, and sister-in-charge, night superintendent, assistant matron, and acting matron of Worcester General Infirmary.

Cottage Hospital, Norwood.—Miss Florence Russell, A.R.R.C., has been appointed matron. She was trained at Westminster Hospital, has been senior sister at Princess Christian's V.A.D. Hospital, Norwood, and as sister in Queen Alexandra's Imperial Military Nursing Service Reserve has served both abroad and at home.

"High Wood," Brentwood, Essex.—Miss M. H. Griffith has been appointed assistant matron. She was trained at Salford Union Infirmary, where she was later staff nurse and sister, has been sister and home sister at the Eastern Fever Hospital, sister at the French Military Hospital, Bordeaux, and theatre sister in hospitals and casualty clearing stations in France.

Birmingham Nerve Hospital.—Mrs. Gladys M. E. Jones has been appointed matron of the in-patient department. She was trained at Guy's Hospital and the Royal Eye Hospital, and has held various appointments as sister-in-charge, night superintendent, matron, etc. She served with the French Red Cross for one year, and at the request of the British Red Cross nursed interned British officers in Murren. A full account of Mrs. Jones' services appeared in our issue of March 29, 1919.

Kingston Union Infirmary.—Miss Anna Sinclair has been appointed matron. She was trained at the Western Infirmary, Glasgow, and took her midwifery training at the Hospital for Women, Brighton, where she filled the post for some time of sister-midwife. She was later sister-in-charge at Fort George Garrison, Inverness, matron of Manor Sanatorium, Peebles, and matron at Great Barr Hospital. During the war Miss Sinclair held the post of matron at various military hospitals.

Royal Albert Hospital, Devonport.—Miss Caroline Webber, R.R.C., has been appointed matron. She was trained at the Coventry and Warwickshire Hospital, where she was afterwards sister, and has been night superintendent at the Radcliffe Infirmary and County Hospital, Oxford. Miss Webber has served two years overseas and two years as matron of the Officers' Hospital, 3rd Southern General Hospital, Oxford.

Boundary Park Hospital, Oldham.—Miss Annie Elizabeth Williams has been appointed charge-nurse. She was trained at Salford Union Infirmary, where she was later staff and charge nurse. She has since been charge nurse at Bridgwater and at Ashton-under-Lyne Union Infirmaries, and sister at 1st Western General Hospital, Western Command, and at the 3rd London General Hospital, Wandsworth.

Blackwood (Mon.), Oakdale Workmen's Hospital.—Miss Hilda Price has been appointed matron. She was trained at Oldham General Infirmary, and has since been sister of the male ward and theatre, Wrexham Infirmary, theatre sister at Bury Infirmary, and at the Jessop Hospital for Women, Sheffield, matron of Ebbw Vale Workmen's Hospital, of the G.W.R. Hospital, Swindon, and of Winchcombe Cottage Hospital. She has also done military nursing.

Wicklow, Newcastle Sanatorium.—Miss Emma Green has been appointed night sister. She was trained at Keighley Union Infirmary.

### THE COLLEGE OF NURSING (Limited by Guarantee).

### LIVERPOOL CENTRE.

Wednesday, January 21, 7 p.m.—A lecture will be given by W. Murray Cairns, Esq., M.D., C.M., in the lecture Theatre of the New Arts Building (first floor), Liverpool University, on "Japan," illustrated by lantern slides. Members of the College will be admitted to the lecture free. Tickets for non-members may be obtained from Miss Golding, 11 Hargreaves Road, Liverpool, price 6d. per lecture.

### LONDON CENTRE.

The members of the London Centre, with their friends, assembled in full strength at the first meeting in the New Year on January 8 to hear Miss Bagley, head of the Polytechnic School of Speech Training, on "Public Speaking." The room of the Medical Society was filled by 8 o'clock, and great disappointment was felt when at 8.15 it was announced that Miss Bagley was unable, owing to illness, to fulfil her engagement. Her address is now to be given at the same time and place on February 12. On the invitation of Miss Biggar, the Chairman of the evening, advantage was taken of the occasion for the members to suggest subjects for general debate at the third meeting of the Series. At first Miss Cowlin was the only one present who appeared to have any suggestion, but after some urging some of the members apparently plucked up courage or sorted out their ideas sufficiently to enable them to suggest propositions for debate, which included such subjects as Equal Pay for Equal Work; Should the Kaiser be Tried? What is Vulgarity? Should the Nurse be Included in the Hours of Employment Bill? Is a Trades Union an Ideal Unit of Organisation for Professional Workers? Is a Sense of Humour Valuable? What Institutional Life is Detrimental to the Development of Character (the last word was amended later to "individuality")? That the State of Ireland is due to Internal Rather than External Factors (a subject which it was suggested might be more vigorously debated if Irish members could be present), etc. On putting these subjects to the vote, that on Institutional Life being Detrimental to the Development of Individuality, was unanimously declared for. It should provide excellent debating matter, and members will look forward to hearing the views of members of their own profession on so vital a point.

### The Unpopularity of District Nursing.

At the instance of Miss Cowlin, an informal discussion on Why So Few Nurses are Attracted to District Nursing then took place, and some interesting side-lights were thrown on the conditions which prevail. Though some suggested that inadequate salaries were the cause of the shortage, the majority attributed it to the conditions of isolation and to a certain extent of discomfort under which the district nurse has often to work. The latter condition was an inevitable consequence of living alone without anyone to clean or cook for the nurse. The liability to calls at any hour of the day and night is another disadvantage in the life, and the consequent impossibility for the conscientious nurse (and no woman should be a district nurse who does not love not only her work but also love people) to get away from her district. It is all these conditions which it was considered cause the trained nurse to hold back from district work. The superintendent of the Berkshire County Nursing Association, which provides a minimum salary of £140 for district nurses and £120 for village nurses, cannot get the nurses she requires. Another speaker suggested that the addition of midwifery to district nursing makes the latter unpopular, and stated she could not get a midwife though a salary of £140 was offered. A suggestion that district nurses should work in pairs so as to provide the necessary relief was vetoed as unpractical and uneconomical.

